# Monocytes/Macrophages in Giant Cell Arteritis-Polymyalgia Rheumatica Spectrum Disease

**DOI:** 10.14336/AD.2025.0830

**Published:** 2026-09-12

**Authors:** Junyan Guo, Yanlin He, Fan Yang, Weiqian Chen

**Affiliations:** ^1^Division of Rheumatology, the First Affiliated Hospital, Zhejiang University School of Medicine, Hangzhou, 310003 Zhejiang, China.; ^2^State Key Laboratory for Diagnosis and Treatment of Infectious Diseases, the First Affiliated Hospital, Zhejiang University School of Medicine, Hangzhou, 310003 Zhejiang, China

**Keywords:** Monocyte, Macrophage, Giant cell arteritis, Polymyalgia rheumatica

## Abstract

Giant cell arteritis (GCA) and polymyalgia rheumatica (PMR) are closely related inflammatory diseases that predominantly affect individuals over 50 years of age. The pathogenesis of two diseases is unclear. Accumulating evidence indicates that aberrant innate and adaptive immune responses underlie the pathogenesis of GCA and PMR. Monocytes/macrophages play an important role in the inflammatory processes through producing proinflammatory cytokines and chemokines, modulating molecular expression, colony-stimulating factors, proteolytic enzymes, and growth factors, and activating the JAK/STAT pathway. Clarifying the functions of monocytes/macrophages may help find targets for these diseases. Current research is investigating potential treatments such as proinflammatory cytokine blockers, anti-CXCR3 agents, competitive antagonists of GM-CSF activity, and JAK inhibitors in patients with GCA or PMR. In this study, we examine the role of monocytes and macrophages in the pathogenesis of GCA and PMR, identify potential drug targets, novel therapeutic strategies and future research directions.

## 1. Background

Giant cell arteritis (GCA) and polymyalgia rheumatica (PMR) are closely related to inflammatory disorders. GCA primarily affects large and medium-sized vessels, with common local symptoms such as acute headache, scalp pain, limb claudication and visual disturbances [1]. PMR is characterized by acute proximal muscle pain and stiffness in the neck, shoulders, upper arms, hips, and thighs [2]. Both GCA and PMR are associated with elevated plasma Interleukin (IL)-6 levels, which contribute to overlapping systemic manifestations, including fever, fatigue, weight loss, depression, night sweats, and elevated inflammatory markers [3]. Approximately 50% of patients with GCA present with PMR at diagnosis or during disease recurrence, while 20% of patients have a history of PMR prior to GCA onset [4]. Their association is supported by clinical, epidemiological and immunological evidence [5], suggesting that GCA and PMR may represent different manifestations of a single disease spectrum. Recent studies propose unifying these conditions under the concept of the GCA-PMR spectrum disease (GPSD) [6].

The pathogenesis of both GCA and PMR is closely related to inflammation. The abnormal activation of innate and adaptive immunity is involved in the pathogenesis of GCA. The key event in GCA pathogenesis is the activation of dendritic cells (DC) via toll-like receptor (TLR) in the arterial wall, which subsequently activate T cells through the expression of co-stimulatory (CD86) and activation (CD83) molecules [7]. Additionally, the expansion of oligoclonal T cells supports antigen-specific adaptive immune responses in GCA [8]. The inflammatory infiltration, vascular wall injury, and remodeling observed in GCA are primarily mediated by macrophages. [9] By contrast, PMR is characterized by synovitis with leukocyte infiltration and angiogenesis, as shown in synovial biopsies [10, 11]. The infiltrating cells are mainly macrophages and memory T cells [11].

Monocytes and macrophages are pivotal in driving inflammatory processes via various immune regulatory mechanisms. These cells are actively involved in all stages of the immune response, from the initiation of inflammation and activation of adaptive immunity to debris clearance and resolution [12]. In the innate immune system, monocytes and macrophages are central components [13]. The activation and polarization of monocytes and macrophages are triggered by recognition of pathogen-associated molecular patterns (PAMPs) and damage-associated molecular patterns (DAMPs) through pattern recognition receptors (PRRs) [14]. Upon PRR engagement, a series of rapid and evolutionarily conserved inflammatory responses are initiated. These responses encompass critical functions such as phagocytosis, cell migration, pathogen or cell elimination, and the production of cytokines, which collectively drive the inflammatory cascade [15].

Given their central role, studying monocyte and macrophages in GCA and PMR is of great importance. These cells exhibit remarkable plasticity: under diverse environmental or pathological contexts, they undergo distinct phenotypic polarization, resulting in different marker expression profiles and functional properties [12]. Understanding these differences can provide insight into how the disease occurs, diagnose the disease, and develop targeted therapies. With the development of scientific and technological means, the classification of monocytes and macrophages has made great progress, which has provided great help for the understanding of diseases. This review summarizes the phenotypes of monocyte as well as macrophages, explores their role in the pathogenesis of GCA and PMR, and discusses potential macrophage-targeted therapeutic.

## 2. Types of monocytes and macrophages

### 2.1 Monocytes

Monocytes, originating from the bone marrow, circulate in the blood before differentiating into macrophages, dendritic cells, and tissue-specific phagocytes. Human monocytes are classified into three subsets: classical monocytes (CD14^++^CD16^-^), which constitute 80-90% of circulating monocytes; intermediate monocytes (CD14^+^ CD16^+^); and non-classical monocytes (CD14^-/low^CD16^+^), making up the remaining 10-20% [16].

**Table 1:** Types of monocytes.

	Classical monocytes	Intermediate monocytes	Non-classical monocytes
**Definition**	CD14^++^CD16^-^	CD14^+^CD16^+^	CD14^-/low^CD16^+^
**CD molecule**	CD64 CD62L	CD86 CD40 CD11b CD74 CD105 CD163	CD86 CD43 CD115 CD294 CD97 CD123
**Cytokine**	IL-6 IL-10 G-CSF	TNF-α IL-1 IL-10	TNF-α IL-1β
**Chemokines and chemokine receptors**	CCR1 CCR2 CXCR1 CXCR2 CCL2	CCR5 CX3CR1	CX3CR1
**Antigen presentation**		HLA-AB HLA-DR	

Classical monocytes are involved in phagocytosis, anti-apoptotic responses, and the response to stimuli essential for wound healing and blood clotting [17, 18]. Classical monocytes, which almost exclusively express C-C motif chemokine receptor 2 (CCR2), C-X-C motif chemokine receptor (CXCR) 1, CXCR2, C-type lectin domain family 4 member D (CLEC4D), IL-13Rα1, and CD62L also produce higher levels of IL-6, C-C motif chemokine ligands (CCL)2, and granulocyte colony-stimulating factor (G-CSF) compared to non-classical and intermediate monocytes [19]. Intermediate monocytes, responsible for T cell proliferation and stimulation, exhibit a high degree of major histocompatibility complex (MHC) Class II processing and antigen presentation [18]. Additionally, they are significant producers of reactive oxygen species (ROS), and the Tie-2^+^ subgroup plays a crucial role in angiogenesis. This subgroup expresses surface markers such as CD202b, CD105, vascular endothelial growth factor receptor 2 (VEGFR2), which are associated with promoting angiogenesis. Although there is debate regarding whether intermediate or non-classical monocytes express the highest levels of MHC Class I molecules, it is clear that CD16^+^ monocytes play a role in activating CD8^+^ T cells [17]. Non-classical monocytes are involved in CD4^+^ T cell proliferation and stimulation [17] and are more likely to induce CD4^+^ T cells to produce IL-4. These monocytes secrete the highest levels of inflammatory cytokines such as tumor necrosis factor-alpha (TNF-α) and IL-1β [18]. Additional information on the different monocyte subtypes is shown in Table 1.

### 2.2 Macrophage

The classic classification of macrophages is M1 and M2 phenotypes: M1 ("classical" activation) and M2 ("alternative" activation). This model is primarily based on *in vitro* data from macrophages stimulated with Type 1 or Type 2 cytokines [20]. In the more recent study, the "M1-like" phenotype is generally described as pro-inflammatory, induced by TLR ligands, interferon-gamma (IFN-γ), and TNF-α. In contrast, "M2-like" macrophages exhibit anti-inflammatory properties, which are activated by interleukin IL-4 or IL-13, and produce transforming growth factor (TGF)-β and other pro-fibrotic factors [21]. Additionally, according to the classification of origin, macrophages can be divided into embryo-derived tissue-resident macrophages and monocyte-derived macrophages.

Macrophages can adopt either pro- and anti-inflammatory properties depending on the disease stage and signals received from the microenvironment. This adaptability, known as macrophage plasticity, enables macrophages to rapidly adjust their phenotype and function in response to changing environmental conditions, a process central to macrophage polarization [22]. However, macrophage plasticity and the spectrum of macrophage activation extend far beyond a simplistic dichotomy of pro- and anti-inflammatory populations. Instead, they encompass a broad and intricate continuum of activated and regulatory phenotypes [12]. Recent advances in single-cell RNA sequencing have revealed that during inflammation, tissue macrophages particularly infiltrating monocyte-derived populations adopt one of four conserved activation states, each associated with distinct functional roles such as phagocytosis, oxidative stress response, inflammation, or tissue remodeling [23]. Notably, embryonically derived macrophages tend to maintain greater transcriptional stability during inflammatory responses, whereas infiltrating monocyte-derived macrophages exhibit significant functional heterogeneity, contributing to the diversity of macrophage responses [24]. These groundbreaking studies are pivotal in redefining the functional spectrum of monocytes and macrophages. These insights have redefined the functional spectrum of monocytes and macrophages, highlighting the need for a revised classification framework.

**Table 2:** Types of macrophages.

Classification mode	Classification						
**Classical classification**	M1	M2					
**Macrophage phenotype and function**	intermediate monocytes	lipid-associated macrophages	tissue-resident macrophages				
**TRM function and** **location**	Kupffer cells	red pulp macrophages	osteoclasts	alveolar macrophages	microglia		
**Tumor associated** **macrophages (TAM)**	inflammatory macrophages	angiogenic macrophages	immune-regulatory macrophages	interferon-mediated regulatory macrophages	immune-stimulating macrophages	CD169 macrophages	
**Single-cell RNA** **sequencing TAM**	interferon-induced TAMs (ifn-TAM)	immunomodulatory TAMs (reg-TAM)	inflammatory cytokine-enriched TAMs (inflammation-TAM)	lipid-related TAMs (la-TAM)	angiogenic TAMs (AngioTAM)	RTM-like TAMs (rtm-TAM)	proliferative TAMs (proliferation-TAM)

Recent studies suggest that the macrophage functional spectrum model, which correlates phenotype with function, is a more accurate framework for describing macrophage subsets [21]. This model builds on the original functional classification of macrophages, which identified subgroups including intermediate monocytes (representing an intermediate stage in macrophage maturation), lipid-associated macrophages (LAMs), and tissue-resident macrophages (TRMs) [25]. The morphology, function, and expression of cell surface markers of TRMs differ significantly across various organs [26]. As tissue resident sentinel phagocytic cells, TRMs help maintain tissue homeostasis, protect blood vessels, and form direct innate defenses against pathogens [27]. Furthermore, TRMs exhibit significant proliferative potential and self-renewal capacity. Bone marrow-derived cells can replenish the macrophage population within inflammatory infiltrates and may replace tissue-resident macrophages of embryonic origin under certain conditions. Additional information on the different macrophages subtypes is shown in Table 2.

## 3 The role of monocytes/macrophages in the pathogenesis of GCA

Monocytes and macrophages are pivotal players in the pathogenesis of large vessel vasculitis. Under both homeostatic and inflammatory conditions, circulating monocytes leave the bloodstream and infiltrate tissues, where they undergo differentiation into macrophages or dendritic cells in response to local growth factors, pro-inflammatory cytokines, and microbial products [28]. *In vivo*, macrophages exert three principal functions: phagocytosis, antigen presentation to exogenous antigens, and immune regulation [29]. Their role in GCA is highlighted in the following aspects:

### 3.1 Monocytes differentiate into macrophages

In patients with GCA, there is an increase in the number of mobilized bone marrow-derived monocytes that shift towards intermediate phenotype [30]. The number of classical monocytes increased in patients with GCA. Classical monocytes express high levels of CCR2 and are recruited from bone marrow to the periphery through the CCL2/CCR2 pathway [31]. The systemic CCL2 levels in GCA patients are reduced, indicating an increased binding of this ligand to classical monocytes, so selectively recruiting these cells [32]. Classical monocytes are regarded as key drivers of inflammation due to their ability to differentiate into tissue macrophages that contribute to tissue lesions [30].

**Figure 1.**
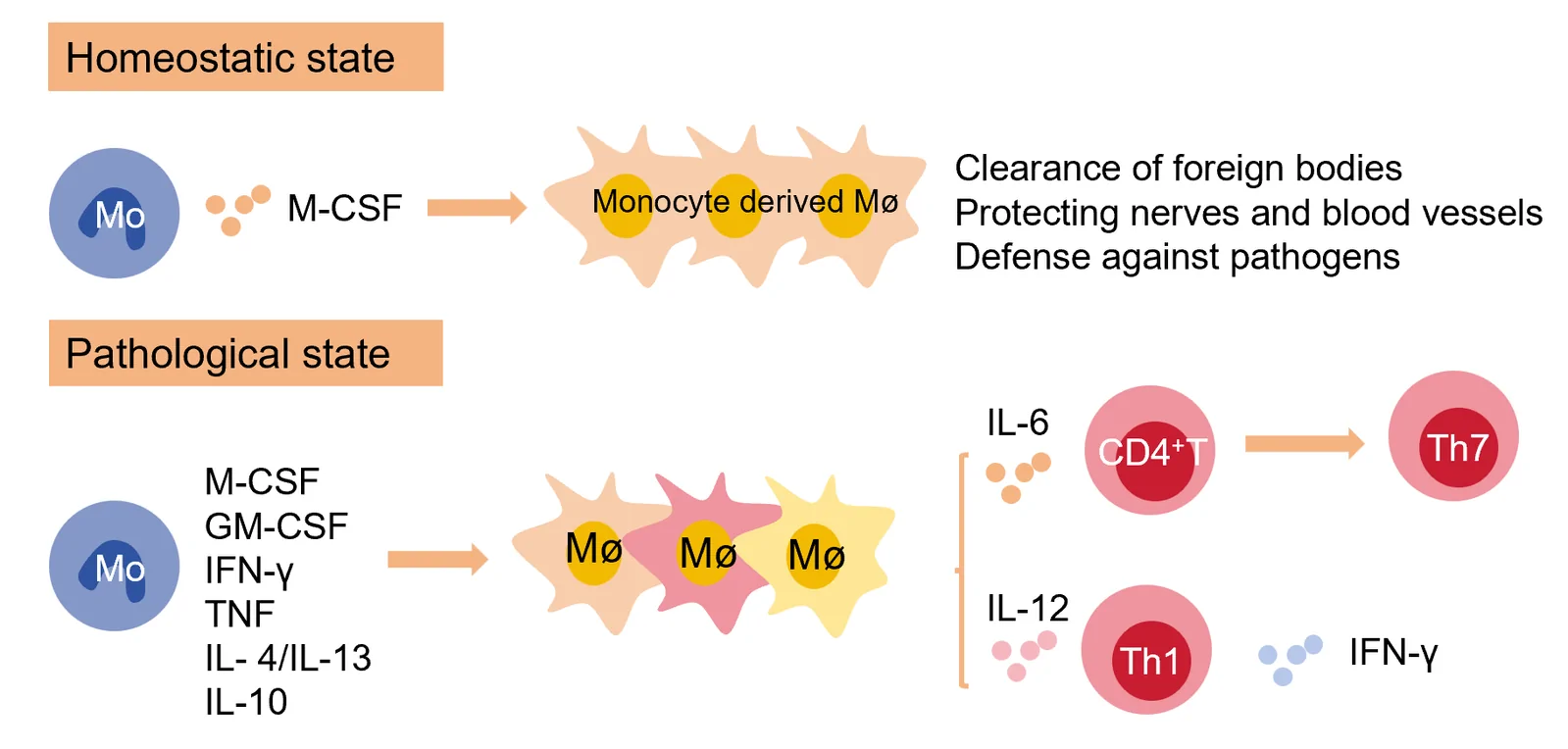
**The monocyte-to-macrophage differentiation path and its influence on T cell polarization**. Mφ, macrophage; Mo, monocytes; T, T cell; Th17, T helper 17 cell; Th1, T helper 1 cell. In homeostatic state, infiltrating monocytes from blood vessels are activated by M-CSF and differentiate into Monocytes derived macrophages. In pathological states, monocytes differentiate into inflammatory macrophages under the influence of various factors. These macrophages produce IL-6 and IL-12. IL-6 promotes the differentiation of naive CD4^+^ T cells into Th17 cells while inhibiting the development of Treg cells. IL-12 serves as the primary inducer of Th1 cell differentiation. Th1 cells release the cytokine IFN-γ.

Although the increase in the number of classical monocytes led to a proportional decrease in non-classical monocytes, non-classical monocytes still play an important role in GCA pathology. Driven by CX3CL1 chemokines, non-classical monocytes infiltrate the arterial wall and develop into inflammatory populations of CD68^+^CD16^+^CX3CR1^+^CCR2^-^ macrophages [32]. Glucocorticoid (GC) can normalize serum CCL2 levels, reduce the number of non-classical monocytes in the blood and the expression of CX3CR1 receptor, reduce monocyte flow into the blood vessel wall, and thus resolve local inflammation [32]. The monocyte-to-macrophage differentiation path and its influence on T cell polarization were shown in Figure 1.

### 3.2 Macrophages regulate proinflammatory cytokines and chemokines

GCA involves two primary clusters of cytokines that influence disease activity: the IL-6/IL-17 axis and the IL-12/IFN-γ axis. These clusters play distinct roles in the vasculitic process, with the IL-6/IL-17 axis being highly responsive to standard GC treatment, while the IL-12/IFN-γ axis shows resistance [33]. CD4^+^ T cells release IFN-γ to activate tissue macrophages [33], and activated macrophages in vascular disease produce IL-6 [34], which promotes differentiation of initial CD4^+^ T cells into T helper 17 cell (Th17) cells while inhibiting differentiation of regulatory T cells (Treg) [35]. Th17 cells release various pro-inflammatory cytokines, including IL-17, IL-21, IL-22, CCL20, granulocyte-macrophage colony-stimulating factor (GM-CSF), IL-8 (CXCL8), and IL-26, which play roles in macrophage and neutrophil recruitment [36, 37]. IL-17 also regulates the recruitment of macrophages and neutrophils [33].

IL-12 is produced by M1 macrophages and is a major inducer of Th1 cells, which release the highly potent cytokine IFN-γ into the microenvironment [33]. IFN-γ controls macrophage activation and regulates disease-related functions of endothelial cells and vascular smooth muscle cells in vasculitis. As an immune amplifier, IFN-γ also induces tissue macrophages to produce chemokines, including IP-10 (CXCL10), Mig (CXCL9), ITAC (CXCL11) [38], and recruits Th1 cells through CXCR3. CCL3, CCL4, and CCL5 are also overproduced from macrophages and recruit T cells via CCR5 [39]. Monocytes are also recruited by chemokines: classical monocytes via the CCL2-CCR2 axis [40] and non-classical monocytes via the CX3CL1-CX3CR1 axis [32]. Tissue macrophages attract both T cells and monocytes/macrophages through various chemokines, thereby amplifying vascular inflammation [41].

Studies have shown that senescent cells are present in patients with GCA and PMR (9.50% and 2.66% respectively) [42]. In GCA patients, p16^+^CD68^+^ and p21^+^CD68^+^senescent macrophages express IL-6 [43], and IL-6(+) senescent cells are associated with the expansion of vascular inflammation [42]. Similarly, a large number of IL-6^+^ cells can be seen in the synovial tissues of middle-aged and elderly patients with PMR. This cytokine has been detected in most CD68^+^ macrophages, indicating that senescent macrophages may produce IL-6 [43]. Non-classical monocytes have short telomeres and exhibit a distinct pro-inflammatory phenotype. Their number increases significantly with age, and they have the characteristics of senescent cells. A reduced proportion of non-classical monocytes was found in the circulation of GCA patients, which may indicate tissue migration of these cells [44].

### 3.3 Macrophages produce colony-stimulating factors and proteolytic enzymes

Macrophages, T cells, myofibroblasts, and endothelial cells in the invaded arteries of GCA patients produce GM-CSF [45]. During the early stages of GCA, infiltrating monocytes from blood vessels are activated by local GM-CSF and differentiate into CD206^+^/matrix metalloproteinase (MMP)-9^+^ macrophages. These macrophages migrate to the meso-intimal junction, where they promote tissue destruction and stimulate angiogenesis through IL-13Rα2 signaling [46]. In the later stage of GCA, CD206^+^/MMP-9^+^ macrophages fuse to form multinuclear giant cells and release M-CSF at the intima-meso boundary [47]. M-CSF is thought to induce macrophages with M2 phenotype [48]. Monocytes/macrophages and giant cells produce excess proteolytic enzymes and proteases, such as collagenase, cathepsin, and matrix metalloproteinase MMP-2 and MMP-9, which damage the elastic membranes and blood vessel walls [49].

### 3.4 Macrophages producing growth factors

Tissue macrophages produce growth factors such as transforming growth factor β1 (TGFβ1), platelet-derived growth factor (PDGF), and fibroblast growth factor (FGF), all of which promote excessive fibroproliferation [41]. Studies of cytokine profiles and localization have shown that, in the early stages of the disease, TGFβ1-producing macrophages are likely to play a role and are activated by IFN-γ released by Th1 cells, although this has not been clearly demonstrated [44]. In the later stages, FRβ^+^ macrophages, biased toward M-CSF signaling at the meso-intimal junction, produce PDGF, which is associated with intimal hyperplasia [50]. Additionally, macrophages at the meso-intimal junction secrete vascular endothelial growth factor (VEGF), influencing the formation of new blood vessels in the outer membrane [51].

### 3.5 Macrophages defectively express checkpoint molecules

The immunosuppressive mechanism of programmed death 1 (PD-L1), which inhibits T cell overactivation, is deficient in GCA vasculopathy. Vascular DCs located in the mesothelial parasite region have defective PD-L1 expression, and these DCs have an increased potential to activate T cells and differentiate initial CD4^+^ T cells into T cells that produce Th1, Th17, and IL-21 [52]. Furthermore, PD-L1 expression was also reduced in monocytes/macrophages in GCA patients [53]. The mechanisms underlying the altered PD-L1 expression on vascular DCs and tissue macrophages require further investigation. Recent studies have also shown that programmed cell death protein 1 (PD-1) pathway may directly regulate both DCs and macrophages, and that PD-1 on macrophages may lead to macrophage dysfunction and incompetence [54]. The lack of PD-L1 may inhibit the binding of the two, thereby slowing the dysfunction of macrophages and allowing the immune response to continue.

## 4. The role of monocytes/macrophages in the pathogenesis of PMR

The pathogenesis of PMR may be associated with several factors, including human leukocyte antigen (HLA), B-cell imbalance, cytotoxic T-lymphocyte-associated protein 4 (CTLA-4) and PD-1, the Janus kinase (JAK)/signal transducer and activator of transcription (STAT) pathway, and abnormal immune responses, particularly T cell abnormalities [55]. In PMR, major congenital immune response disorders are characterized by neutrophil and monocyte overactivity, expression of TLR7 in active disease, impaired phagocytosis, endothelial dysfunction, and an increased innate T cell count in patients in remission [56]. In PMR patients, an increase in classical monocytes and a decrease in CCL2 levels were also observed [32]. Abnormal adaptive immune responses are evidenced by the polarization of Th1 and Th17 phenotypes in tissues and serum, elevated expression of immune aging markers, and downregulation of immunomodulatory responses. Changes in the distribution of peripheral B cells have also been detected during active disease, indicating their migration to an unknown site in the periphery [56].

The interaction between innate and adaptive immune responses has been confirmed by synovial infiltration of macrophages and T cells. Studies have shown that immune cells infiltrating the muscle and joint areas. Arthroscopic biopsies from PMR patients with shoulder synovitis revealed macrophage and T cell infiltration, without B cells, NK cells, or gamma/delta T cells present [11].

In terms of cytokine profiles, IL-6 plays a central role, but other pro-inflammatory mediators and angiogenic markers, such as chemokines, B-cell activating factors, VEGF, and angiopoietin, are also implicated in refractory or GC-resistant PMR [56]. Many studies have demonstrated significantly elevated circulating IL-6 levels in patients with active GCA or PMR [57]. IL-6 mediates its effects through two distinct pathways: the classical and trans-signaling pathways. In classical signaling, IL-6 binds to membrane-bound IL-6R, activating gp130 and initiating intracellular cascades in monocytes, macrophages, hepatocytes, certain lymphocytes, megakaryocytes, and endothelial cells. Stimulation of hepatocyte leads to increased production of acute-phase proteins such as CRP which is crucial characteristic of active GCA and PMR. Trans-signaling occurs when IL-6 binds to soluble IL-6R, forming a complex that activates membrane-bound gp130. Additionally, IL-6 regulates the Th17/Treg imbalance in active GCA, playing crucial roles in immune regulation [58]. Nevertheless, the correlation between circulating IL-6 levels and acute-phase markers (ESR, CRP, platelet count) in GCA and PMR remains controversial [57]. Metabolic studies reveal that ESR and CRP not only correlate strongly with GlycA/GlycB during disease activity but also associate with ketone bodies (3-hydroxybutyric acid and acetone). Notably, 3-hydroxybutyric acid elevation was observed in PMR but not GCA patients after 6 months of treatment [59].

There is no consensus on the exact source of IL-6. It has been suggested that CD14^+^ cells (monocytes) in peripheral blood are primarily responsible for its production [3]. Cytokine levels are elevated in both peripheral blood and tissue samples from PMR patients, with most being produced by innate immune cells. However, mononuclear cells extracted from PMR patient peripheral blood did not show a direct correlation with IL-6 production after cell stimulation in culture [60]. TLR7 activation may occur during active PMR, with its activity potentially decreasing during disease remission [61]. This may explain the activation of the immune system and IL-6 production in PMR or could simply reflect the intense inflammatory activity observed in the disease [62].

## 5. Similarities and differences in the role of monocytes/macrophages in the pathogenesis of GCA and PMR

Based on the aforementioned evidence, monocytes and macrophages play overlapping roles in the pathogenesis of both GCA and PMR. In both diseases, monocytes are recruited to sites of inflammation through chemokine-mediated pathways. Specifically, classical monocytes and non-classical monocytes are mobilized by chemokines such as CCL2/CCR2 and CX3CL1/CX3CR1, respectively. Upon reaching the inflammatory sites, these monocytes differentiate into macrophages and contribute to local inflammatory responses. In both GCA and PMR, monocytes/macrophages exacerbate inflammation by producing pro-inflammatory cytokines, including IL-6, IL-12, and IFN-γ, which promote the differentiation of Th1 and Th17 cells. Additionally, defective expression of PD-L1 in monocytes/macrophages in both GCA and PMR leads to excessive T cell activation, further amplifying the inflammatory cascade. Macrophages in both conditions also produce growth factors such as TGFβ1, PDGF, and VEGF, which contribute to fibroproliferation and neovascularization. The role of monocyte/macrophages in GCA/PMR has been summarized in Table 3.

**Table 3:** The role of monocytes/macrophages in GCA/PMR.

	GCA	PMR
**Monocyte**	1. Classic monocytes were increase [32], non-classical and intermediate monocytes were unchanged. 2. Classic monocytes expressed high CCR2 and low CX3CR1, non-classical monocytes expressed CX3CR1, lack CCR2. 3. Non-classical monocytes shared the similar phenotype with tissue CD68^+^CD16^+^CX3CR1^+^CCR2^-^ macrophages [32]. 4. Circulating monocytes were prone to produce MMP-2 and MMP-9 [102].	1. Classic monocytes were increase [32,103], or decreased [104]; non-classical and intermediate monocytes were unchanged. 2. Classic monocytes expressed high CCR2 and low CX3CR1, non-classical monocytes expressed CX3CR1, lack CCR2. 3. Monocytes produce IL-6 [56].
**Macrophage**	CD68^+^CD16^+^CX3CR1^+^CCR2^-^ macrophages were founded in GCA temporal artery vessel tissues [32]. CD206^+^/MMP-9^+^ macrophages were located at the site of tissue destruction, whereas FRβ^+^macrophages were located in the inner intima of GCA arteries [46, 50]. Macrophages producing IL-6, which promotes differentiation of initial CD4^+^ T cells into Th17 cells while inhibiting differentiation of Treg [34, 35]. M1 macrophages secrete reactive oxygen species intermediates and matrix metalloproteinases, which damage the arterial wall by destroying endothelial cells and vascular smooth muscle cells [105]. M2 macrophages release angiogenic growth factors such as VEGF, FGF, and PDGF, leading to thickening of the arterial wall and vascular occlusion [105].	1. Proinflammatory macrophages were found in PMR bursa tissue [103]. 2. Macrophages produce pro-inflammatory cytokines (including IL-6, IL-12 and IFN-γ), exacerbating inflammation[103], promoting the differentiation of Th1 and Th17 cells [56].

Despite these similarities, monocytes/macrophages exhibit distinct roles in GCA and PMR, primarily due to differences in the sites of infiltration and disease-specific pathological mechanisms. In GCA, both the IL-6/IL-17 axis and the IL-12/IFN-γ axis play critical roles in disease progression. Notably, the IL-12/IFN-γ axis is resistant to GC therapy, which makes the treatment for some GCA patients more complicated [33]. In contrast, PMR is predominantly driven by the IL-6 axis, with other pro-inflammatory mediators, such as VEGF and chemokines, also contributing to disease progression. This difference in cytokine dominance may explain why PMR patients generally exhibit greater sensitivity to GC therapy, although some may develop GC dependence or resistance over time. Pathologically, GCA primarily affects large blood vessels, particularly the temporal arteries, and is characterized by monocyte/macrophage infiltration, giant cell formation, and vascular wall destruction. In contrast, PMR mainly involves joints and muscles, with pathological features including monocyte/macrophage and T cell infiltration in synovial and muscle tissues. Furthermore, in GCA, monocytes are activated by local GM-CSF and differentiate into CD206^+^/MMP-9^+^ macrophages, which play a critical role in vascular wall destruction and fibroproliferation. To date, no similar pathological mechanism involving GM-CSF-driven macrophage differentiation has been reported in PMR.

## 6. Potential Targets in Monocytes and Macrophages

**Figure 2.**
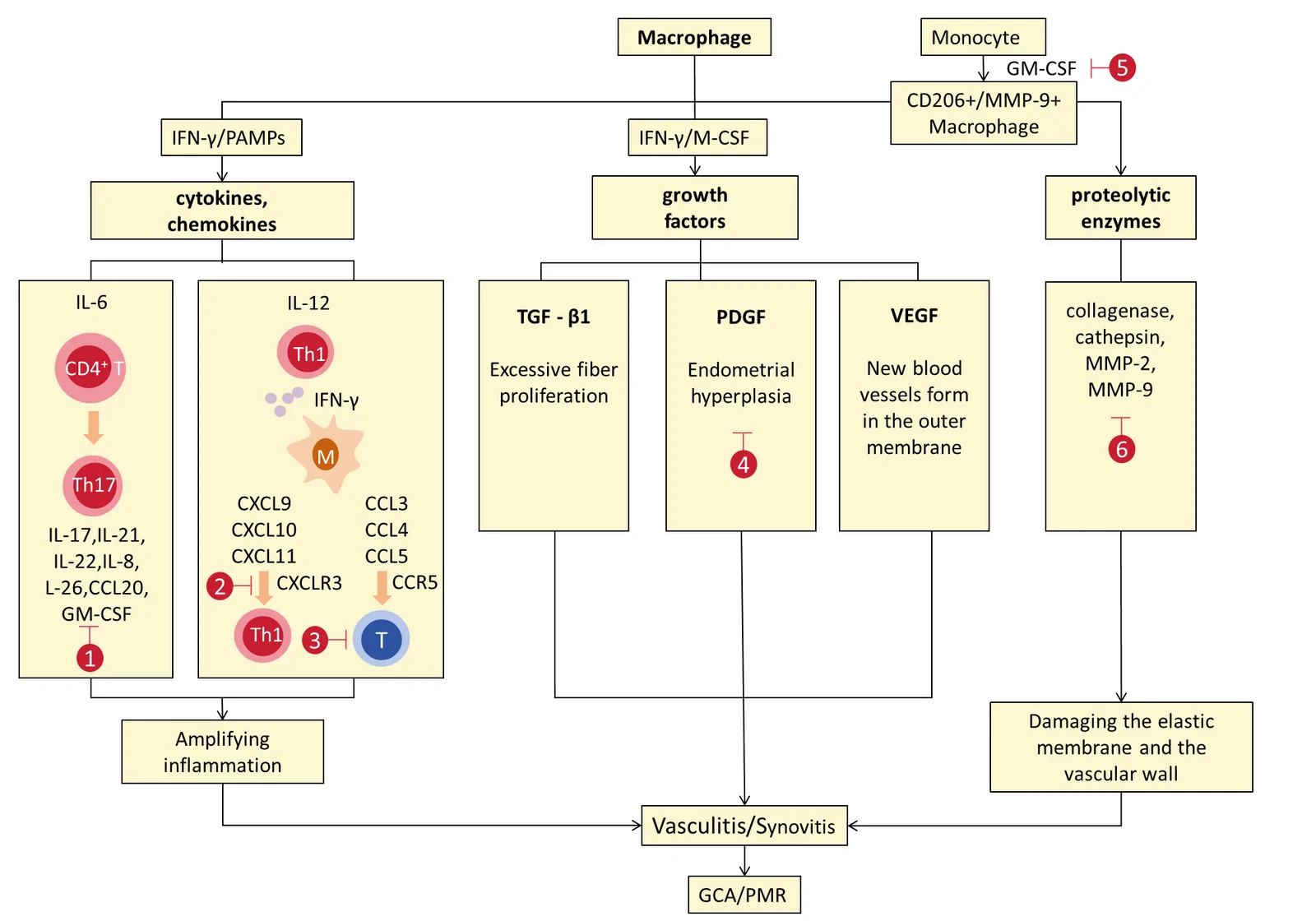
**The potential therapeutic targets of monocytes/macrophages in GCA and PMR**. M, macrophage; T, T cells; 1, Proinflammatory cytokines receptor antagonist; 2, Anti-CXCR3; 3, T-cell inhibitor; 4, Anti-PDGF; 5, Anti-GM-CSF receptor; 6, MMP-9 Inhibitors. Monocytes/macrophages cause GCA/PMR through different pathways and provide potential therapeutic targets. JAK Inhibitors acts on multiple pathways.

Monocytes and macrophages are pivotal in the pathogenesis of GCA and PMR. Targeting these cells may offer novel therapeutic strategies. The recent treatment of GCA and PMR is primarily based on oral GC. Intramuscular methylprednisolone may serve as an alternative for PMR patients [63]. Methotrexate (MTX) has been shown to reduce the cumulative dose and recurrence rate of GC in GCA [64], and evidence suggests that it is also beneficial for PMR patients [65]. Azathioprine trials in GCA have yielded some positive results [66], though data for PMR remain limited. Biologic disease-modifying antirheumatic drugs (DMARDs), such as the monoclonal anti-IL-6 receptor antibody (tocilizumab), have proven effective in treating GCA [67]. In GCA patients, the addition of tocilizumab leads to a higher response rate compared to GC monotherapy [68, 69]. Although data on tocilizumab for PMR are sparse, case reports and series suggest that intravenous tocilizumab may be effective in individual PMR patients [70]. ABA (abatacept), a fusion protein that blocks T-cell co-stimulation, has also shown promise in reducing relapse risk in a small GCA trial [71]. More recently, a Phase 3 clinical trial demonstrated that Upadacitinib, a JAK inhibitor, combined with 26-week GC in GCA patients gradually reduced GC dosage and demonstrated a favorable benefit-risk profile [72]. And these new drugs are highly likely to be used alongside tocilizumab as a first-line treatment [73]. The following content summarizes the potential therapeutic targets of monocytes/macrophages in GCA and PMR (Fig. 2).

### 6.1 Proinflammatory Cytokines

The GiACTA trial has successfully demonstrated that blocking IL-6 signaling with tocilizumab (TCZ) can prevent GCA onset and reduce corticosteroid use [69]. However, relapses are still common after discontinuation. TCZ can be used to manage patients who experience relapse [74]. Anti-TNF-α has shown different results in GCA treatment [75]. Despite initial case reports demonstrating the efficacy of anti-TNF-α therapy in GCA, prospective clinical trials have not shown significant benefits [76]. In the first randomized trial, infliximab failed to reduce relapse rates at 22 weeks or during GC tapering to 10 mg/day compared to placebo, while also increasing infection risk. A small double-blind trial (n=17) evaluating etanercept was inconclusive due to limited sample size [77]. Similarly, a larger RCT (n=70) found that adalimumab neither reduced prednisone requirements nor prevented relapse [78]. These findings suggest that while TNF-α may not contribute to GCA inflammation, its role can likely be compensated by other cytokines, making targeted blockade insufficient for disease control [79]. The discrepancy between case reports and clinical trials may stem from differences in drug selection, sample size, and disease severity—with case reports typically involving fewer, more refractory patients who poorly respond to GC therapy. Anakinra, an IL-1 receptor antagonist, has shown some efficacy in case series [80], but its overall effectiveness and safety in large-scale trials remain unproven. Ustekinumab (UST), a human immunoglobulin G1 kappa (IgG1 κ) monoclonal antibody targeting the shared p40 subunit of IL-12 and IL-23, has shown potential in blocking Th1 and Th17 responses [81-83], though contradictory research results suggest more experimental validation is needed [84]. An open-label, single-arm study in relapsing GCA patients suggested potential clinical benefits of UST [85]. However, a subsequent small open-label trial found that UST combined with 24 weeks of prednisone led to a high treatment failure rate [86]. The role of IL-12/23p40 in GCA remains unclear, as it is minimally express—or even undetectable—in some patients. Given the complexity of this pathway, where dynamic subunit combinations may exert opposing effects, biological interference could yield unpredictable outcomes, potentially explaining the inconsistent trial results [79]. Larger randomized controlled trials are needed to definitively assess UST’s efficacy in GCA. Secukinumab, a selective monoclonal antibody targeting IL-17A, has demonstrated that patients with active GCA achieved a higher rate of sustained remission in the secukinumab group compared to the placebo group when combined with a GC tapering regimen [87]. Other clinical trials under investigation for secukinumab treatment of GCS or PMR are in Phase III (NCT04930094, NCT05380453, NCT05767034).

### 6.2 JAK Inhibitors

The therapeutic efficacy of a single target is limited, and combinations of drugs that inhibit multiple cell signals, such as JAK inhibitors, may be effective therapeutically [41]. Multiple cytokines involved in the pathogenesis of GCA, including IL-6, IFN-γ, IFN-α, and GM-CSF, utilize the JAK-STAT pathway [88]. Animal models of large vessel vasculitis show that JAK inhibitors can reduce T cell infiltration, cytokine production, and macrophage activity, thus decreasing neovascularization and intimal hyperplasia [89]. JAK inhibitor could be effective in GCA according to a real-world analysis and review [90]. An open-label randomized controlled trial indicates that the efficacy and safety of the JAK inhibitor tofacitinib in patients with PMR are not statistically significantly different from those of GC [91]. More promisingly, a phase III RCT of upadacitinib combined with 26-week GC in GCA patients demonstrated superior efficacy and reduced GC use [72].

### 6.3 T Cell Regulation

T cell responses, particularly those mediated by the Th1 and Th17 subsets, play a central role in the pathogenesis of both GCA and PMR. ABA is a CTLA4-Ig small molecule fusion protein that binds to CD80/CD86, thereby preventing CD28 from binding to its ligand and ultimately inhibiting T cell activation [92]. But the results of existing studies have been inconsistent. A randomized, double-blind trial demonstrated that the treatment regimen combining prednisone and ABA significantly reduced the risk of relapse in patients with GCA [71]. Another study compared the efficacy of ABA to TCZ, concluding that both agents are effective therapeutic options for GCA [93]. However, a meta-analysis indicated that abatacept did not show superior efficacy compared to placebo [94]. With respect to PMR, available data suggested that abatacept monotherapy may not provide sufficient benefit to justify large-scale trials in early-stage PMR [95]. Overall, the response of abatacept in GCA was better than that in PMR. The differences in the conclusions of the above experiments may stem from different experimental protocols, including treatment cycles and the combined use of GC [79].

### 6.4 Anti-CXCR3

Targeting chemokine and chemokine receptor interactions (eg. CXCR3) to prevent the recruitment of circulating monocytes and T cells holds promise. However, once clinical symptoms appear, large numbers of monocytes are already recruited to the vascular tissue, where differentiated macrophages are resistant to current therapies, limiting the effectiveness of chemokine blockade. Therefore, the clinical benefits of blocking chemokine receptor interactions are unclear, and although some animal trials of this approach to treat rheumatic diseases have been effective, clinical trials have been mostly unsuccessful [96].

### 6.5 Anti-GM-CSF receptor

Both GM-CSF and its receptor, GM-CSF-α, are up-regulated in the temporal artery biopsy (TAB) of patients with GCA and elevated levels of GM-CSF have also been observed in the peripheral blood of patients with active GCA [84]. Mavrilimumab (MAV) is an IgG4 humanized monoclonal antibody that inhibits GM-CSF receptor alpha and is a competitive antagonist of GM-CSF activity. Studies have shown that MAV can reduce infiltrating cells, pro-inflammatory markers and new angiogenesis in cultured arteries of GCA patients, because it decreased the expression of molecules associated with T cell activation (HLA-DR) and Th1 differentiation (IFN-γ), pro-inflammatory cytokines IL-6, TNF-α, and IL-1β, and molecules associated with vascular damage (MMP-9, lipid peroxidation products, and inducible nitric oxide synthase) [45]. A phase 2 RCT further indicated that MAV was superior to placebo in prolonging time to flare by week 26 and in achieving sustained remission in patients with GCA [97]. However, the existing experiments have merely demonstrated the *in vitro* effects or clinical responses of this target. More human data and experiments are needed to prove its effects *in vivo*.

### 6.6 Targeted Vascular Remodeling

Vascular remodeling, a key driver of occlusion and ischemic complications in GCA, is insufficiently controlled by GCs, which primarily target inflammatory pathways [79]. However, GCs exert limited effects on vascular remodeling factors such as TGF-β, PDGF, and endothelin-1 [79]. Findings from experimental GCA models suggest that blocking PDGF receptor signaling with imatinib or neutralizing endothelin-1 receptors can inhibit vascular smooth muscle cell (VSMC) responses and mitigate vascular remodeling [98, 99].

### 6.7 PD-L1 Fc

Monocytes in GCA and PMR show reduced PD-L1 expression. Low PD-L1 expression leads to an increased ability of monocytes to stimulate, which contributes to overactivation of CD4^+^T cells [100]. This defect arises from increased lysosomal degradation of PD-L1 [101]. A study of Antineutrophil cytoplasmic antibody (ANCA) -associated vasculitis shows that correcting this defect by targeting lysosomal function can inhibit overactivated T cells in induced vasculitis and improve vascular inflammation [101]. However, no studies to date have investigated the therapeutic potential of PD-L1 Fc in GCA. More laboratory research and clinical studies are needed to verify the effectiveness of this target.

## Conclusion

This review highlights the pivotal role of monocytes and macrophages in the pathogenesis of the GCA-PMR spectrum disease (GPSD) and explores potential therapeutic targets. Pathogenic mechanisms involve the secretion of proinflammatory cytokines and chemokines, modulation of molecular expression, production of colony-stimulating factors, release of proteolytic enzymes and growth factors, and activation of the JAK/STAT signaling pathway. Against the above targets, current research is exploring several potential approaches, including inhibitors of proinflammatory cytokines, anti-CXCR3 agents, PD-L1 Fc fusion proteins, competitive antagonists of GM-CSF activity, and JAK inhibitors. Although many targets do not yet have drugs or new drugs in vitro or animal trials, mononuclear macrophage-related pathways still provide new ideas for the treatment of GCA and PMR. Future studies are needed to delineate how monocyte/macrophage profiles differ across GPSD phenotypes and how these differences may influence therapeutic response.
